# Validation of the Turkish adaptation of FACETS-OF-PPC: a multidimensional outcome measure for pediatric palliative care

**DOI:** 10.3389/fonc.2025.1510099

**Published:** 2025-04-11

**Authors:** Fatma Zehra Oztek Celebi, Yasemin Bozdag, Songul Deniz Boybeyi, Melahat Melek Oguz, Esma Altinel Acoglu, Saliha Senel, Sanliay Sahin

**Affiliations:** ^1^ Department of Pediatrics, Dr. Sami Ulus Maternity and Pediatric Health and Disease Training and Research Hospital, Ankara, Türkiye; ^2^ Department of Pediatrics, University of Health Sciences, Istanbul, Türkiye; ^3^ Department of Pediatrics, Faculty of Medicine, Ankara Yildirim Beyazit University, Ankara, Türkiye

**Keywords:** children, cultural adaptation of outcome assessment, life limiting disease, life treating disease, neurological impairment, palliative care standards

## Abstract

**Introduction:**

This study aims to validate the Turkish version of the Family-Centered Multidimensional Outcome Measure for Pediatric Palliative Care (FACETS-OF-PPC), originally developed in Germany for children with severe neurological impairments and their families.

**Methods:**

The FACETS-OF-PPC was translated and culturally adapted following the World Health Organization's guidelines. Following expert reviews and pilot testing, the final version was completed and implemented between February and December 2021 at a pediatric palliative care center in Türkiye. Participants included family members and healthcare professionals closely involved with the patients. Exclusion criteria were age over 18, end-of-life stage, or non-Turkish speakers. Confirmatory factor analysis was conducted to evaluate the factorial validity.

**Results and discussion:**

The study analyzed 102 responses (51 parents, 51 healthcare professionals), revealing suboptimal model fit (X^2^/df = 2.29; CFI=0.805; TLI=0.757; SRMR=0.109; RMSEA = 0.114). Internal consistency was adequate for the "normalcy" (w = 0.87) and "caregiver competencies" (w = 0.86) scales, but insufficient for "child’s social participation" (w = 0.51), "social support" (w = 0.20), and "coping with the disease" (w = 0.50). While the Turkish version of FACETS-OF-PPC showed reliable results for certain dimensions, cultural differences and the small sample size likely affected the overall validity, suggesting the need for further refinement.

## Introduction

Palliative care (PC) is a holistic approach that is emerged initially for oncology patients. Currently it has become widespread for all patients with life threating and limiting diseases. The modern PC movement, which began with the pioneering work of Dame Cicely Saunders, has since evolved and expanded in many western countries. The classification system for PC, based on indicators like service availability, policies, opioid use, and integration, categorized Türkiye under Category 3a (1). This level means that PC has been available in a few areas and is not fully integrated into the national healthcare system (2). The organization of PC can be evaluated in 3 groups: inpatient PC services, hospital support teams, and home care teams (3). The first formal attempt to establish PC in Turkey was made with the Pallia-Turk project. The main priorities of the project were the development of opioid availability and a community-based model of PC. In 2014, the Ministry of Health published guidelines for PC services, leading to a rise in newly registered inpatient PC units (4–6). The first pediatric palliative care (PPC) center in Türkiye was opened in 2015 (7, 8). In 2024, there are 13 inpatient PPC services, no hospital support teams and limited home care teams for children with life limiting and treating diseases in Türkiye.

Measuring outcomes, particularly those reported by patients, is receiving increasing focus in PC as a means to evaluate the quality, effectiveness, efficiency, and accessibility of care. The European Association for Palliative Care (EAPC) Task Force on Outcome Measurement provides key recommendations on the use, selection, and implementation of outcome measures, as well as on utilizing these outcomes for national and international comparisons and benchmarking in clinical practice and research (9). Additionally, the GO-PPaCS (Global Overview – Pediatric Palliative Care Standards) project, an initiative to redefine international PPC standards, highlights the need for culturally and contextually validated tools to ensure reliable and applicable outcome measurements in diverse settings (10). Such measurement is essential for understanding care models, patient complexity, and the broader impact of PC. Outcome measures that are equally essential in PPC also have to be multidimensional to capture fully the holistic nature of PPC: physical, psychological, social, and spiritual aspects (9–11).

There has been little research specifically addressing multidimensional outcome measurement in PPC for patients with congenital and neurological conditions. Although these children represent the largest patient group in PPC (12), the majority of the studies on multidimensional outcome measurement have primarily concentrated on children with cancer. To address this gap, Pelke et al. developed the FACETS-OF-PPC (Family-Centered Multidimensional Outcome Measure for Pediatric Palliative Care), a family-centered multidimensional tool designed specifically for children with severe neurological impairments and their families (13). This tool takes into account the entire care unit, considering both the child’s and the family’s needs. Importantly, FACETS-OF-PPC has been validated in Germany, emphasizing its relevance and applicability in clinical practice (11).

The EAPC Task Force on Outcome Measurement recommends using measures that enable comparisons across different care settings and throughout Europe. Therefore, PC centers should adopt measures that are culturally sensitive and have validated translations for the relevant languages and countries (9). Following this recommendation, this study aims to validate the Turkish version of the FACETS-OF-PPC.

## Materials and methods

### Design

A cross-sectional, single-center study was undertaken to evaluate the Turkish version of the FACETS-OF-PPC. In 2020, Dr. Oztek Celebi attended the PPC training program in Germany, where she collaborated with the FACETS-OF-PPC research team. Together, they initiated the validation process for the Turkish adaptation of the FACETS-OF-PPC.

### Translation and adaptation of the FACETS-OF-PPC into Turkish

This study is a cross-sectional validity and reliability study. The German-validated FACETS-OF-PPC was applied to Turkish PPC patients living in Türkiye to test its validity and reliability. The translation and adaptation process followed the guidelines published by the World Health Organization for scale adaptation (14). The tool was independently translated into Turkish by Dr. Oztek Celebi and a certified translator; Dr. Oztek Celebi, a native Turkish speaker fluent in German, led the translation. After five experienced physicians reviewed it, further revisions were made to improve clarity. The back-translation into German was done by Dr. Bozdag, a bilingual pediatric resident. A comparison of the back-translated version with the original scale showed only minor differences. Since there were limited PPC patients available, the tool was piloted with parents of patients admitted to the hospital for pneumonia. Feedback provided by these parents was carefully analyzed, and revisions were made to address any areas of ambiguity. The final version was then formalized.

### Measures

The FACETS-OF-PPC consists of 39 items, with 34 organized into six subscales: “symptoms,” “child’s social participation,” “normalcy,” “social support,” “coping with the disease,” and “caregiver’s competencies.” (11). The remaining five items focus on additional aspects such as other symptoms, the parent’s partner, and the ill child’s siblings. The majority of the items are scored on a 6-point Likert scale, ranging from 1 (completely disagree) to 6 (completely agree), reflecting experiences during the past seven days. Symptom severity is rated from 1 (not present) to 6 (very pronounced). Separate versions of the questionnaire were provided for parents and professional caregivers (11). Pelke et al. (11) reduced the original 34 items to 17 after stepwise refinement to improve the model’s statistical fit. The final 17 items were grouped into five scales—child’s social participation, normalcy, social support, coping with the disease, and caregiver competencies (see **Table 1**) —each demonstrating sufficient internal consistency for reliable scoring (11). We used 39 itemed original version of FACETS-OF-PPC. In accordance with the methodology used by Pelke et al. (11), we used 17 items for the factor analysis. The symptom burden including secretions, respiratory distress, agitation, pain, sleep problems, convulsions, and spasticity was assessed independently. A score was calculated for each patient across five scales: child’s social participation, normalcy, social support, coping with the disease, and caregiver competencies. This calculation was performed by summing the numbers from 1 to 6 on the Likert scale for each item in the respective scale and dividing the total by the number of items in that scale. To ensure accurate results, items 9, 10, 12, and 14 from **Table 1** were reverse-coded.

**Table 1:** Questionnaire scales and their respective items.

Scale	Items
Child’s social participation	1. My child took part in social life according to his/her abilities.
	2. I have ideas on how to keep my child occupied in daily life.
	3. Besides his/her limitations, my child also has abilities.
Normalcy	4. I had time to do the things that make me happy.
	5. I had time to myself.
	6. Despite my child’s illness, I was able to maintain social contacts.
	7. My everyday life was predictable.
	8. A normal family life was possible for us.
Social support	9. I was alone in dealing with my child’s illness.
	10. I was alone with my grief.
	11. I could talk openly about my child’s illness in my social environment.
Coping with the disease	12. I despair at the question of why my child is affected.
	13. I can accept my child’s illness.
	14. I feel guilty for my child’s illness.
Caregiver’s competencies	15. I am prepared for my child’s crises.
	16. If necessary, I am able to independently take measures to alleviate my child’s symptoms.
	17. I have a clear idea of what should be done for my child in a medical emergency.

### Recruitment and data collection

The data collection was conducted between February and December 2021, using the finalized tool, with families of severely disabled and non-verbal patients who were either hospitalized in the PPC unit of Dr. Sami Ulus Maternity and Pediatric Health and Disease Training and Research Hospital or receiving home healthcare from the same institution, after obtaining their written consent. Healthcare professionals familiar with the patient and family also completed the professional version of the tool, with both their own and the family’s written consent. Additionally, demographic data were collected from both groups, and an evaluation form was provided, asking participants to highlight any questions they found difficult or uncomfortable. The study included patients aged 1 month to 18 years who had been receiving care from the PC Unit or Home Healthcare Services for at least three months. Patients over 18 years of age, those in the end-of-life stage, and non-native Turkish speakers were excluded from the study.

### Statistical analysis

The statistical analysis of the data was performed using SPSS version 20.0. Frequency and percentage values were provided for nominal variables, while mean and standard deviation values were given for continuous variables. The Student’s t-test was used to compare the symptom burden and five scales between family and healthcare professional assessments. The evaluation form and the demographic questionnaire were analyzed descriptively. Values of P<0.05 were considered statistically significant.

A CFA was performed on the parent and professional caregiver data using the lavaan (15) package in R to evaluate the factorial validity of the Turkish version of the FACETS-OF-PPC. The model fit was interpreted according to the recommendations provided by Schreiber (16) chi-square to degrees of freedom ratio (X²/df) (<3 = acceptable, <2 = good). Additionally, the Comparative Fit Index (CFI: ≥0.95 = acceptable), Tucker-Lewis Index (TLI ≥ 0.95 = good), Standardized Root Mean Square Residual (SRMR ≤ 0.08 = good), and Root Mean Square Error of Approximation (RMSEA, <0.08 = acceptable; <0.06 = good) were used to evaluate the model’s adequacy (17).

McDonald’s ω was computed to assess internal consistency, with values between 0.7 and 0.9 considered ideal. This approach was chosen over the commonly used Cronbach’s α, which assumes strict τ-equivalency (18).

### Ethical approval and informed consent

This study received approval from the Ethics Committee of Dr. Sami Ulus Maternity and Pediatric Health and Disease Training and Research Hospital (ID: E-21/01-73) and was carried out in line with the ethical principles outlined in the Declaration of Helsinki.

## Results

### Demographic data and descriptive analysis of FACETS-OF-PPC

Overall, 51 parents of 51 children and 51 professional caregivers were recruited for study participation. **Table 1** shows the characteristics of patients, their parents and their professional caregivers. Of the children, 39 (76.5%) were receiving some form of respiratory support, including oxygen therapy, non-invasive, or invasive mechanical ventilation. Additionally, 44 (86.3%) patients required nutritional support, which included oral enteral nutrition or feeding through naso-gastric or gastrostomy tubes (See **Table 2**).

**Table 2:** Characteristics of the parents, their children and professional caregivers.

Parents	
Study participants; n (%)	
• Mother	46 (90.2%)
• Father	3 (5.9%)
• Other	2 (3.9%)
Parents’ age in years; Mean (SD)	
• Mothers	33.5 (7.1)
• Fathers	37.4 (7.3)
Mothers’ educational status; n (%)	
• Illiterate	2 (3.9%)
• Primary school	11 (21.6%)
• Secondary school	16 (31.4%)
• High school	14 (27.5%)
• University	7 (13.7%)
Fathers’ educational status; n (%)	
• Illiterate	0
• Primary school	12 (23.5%)
• Secondary school	14 (27.5%)
• High school	17 (33.3%)
• University	6 (11.8%)
Children	
Child’s sex; n (%)	
• Male	31 (60.8%)
• Female	20 (39.2%)
Child’s age in years; Mean (SD)	6.4 (4.9)
Child’s diagnosis; n (%)	
• Metabolic diseases	9 (17.6%)
• Diseases of the nervous system	36 (70.6%)
• Syndromes	1 (2.0%)
• Congenital heart disaese	2 (3.9%)
• Missing	3 (5.9%)
Child’s status of respiratory support; n (%)	
• No respiratory support	11 (21.6%)
• Respiratory support with oxygen	3 (5.9%)
• Respiratory support with non-invasiv mechanical ventilation	4 (7.8%)
• Respiratory support with home-type mechanical ventilation	32 (62.7%)
• Missing	1 (2.0%)
Child’s status of nutritional support; n (%)	
• No nutritional support	5 (9.8%)
• Enteral nutrition via oral intake	5 (9.8%)
• Nutrinal support via nasogastric tube	22 (43.1%)
• Nutritional support via gastrostomy tube	17 (33.3%)
• Missing	2 (3.9%)
Child’s number of siblings; n (%)	
• 0	15 (29.4%)
• 1	19 (37.3%)
• 2	11 (21.6%)
• 3	3 (5.9%)
• Missing	3 (5.9%)
Child’s own room; n (%)	
• Yes	33 (64.7%)
• No	16 (31.4%)
• Missing	2 (3.9%)
Sample; n (%)	
• Inpatient	36 (70.6%)
• Outpatient	15 (29.4%)
Child’s care provider; n (%)	
Mother	51 (100)
Healthcare professional	
Sex (m/f)	1/50
Age in years; M (SD)	36.7 (8.6)
Work experience in PC in years d; n (%)	
• 0–1	27 (52.9%)
• >1–2	15 (29.4%)
• >2–5	9 (17.7%)
Work setting; n (%)	
• Pediatric Palliative Care Unit	36 (70.6%)
• Home health services	15 (29.4%)
Profession e; n (%)	
• Physician	27 (52.9%)
• Nurse	24 (47.1%)
The duration of care provided by the healthcare professional to the patient in months, median (min-max)	8 (1-36)

Parents and professional caregivers assessed the current version of the FACETS-OF-PPC as appropriate in length, easily understandable, containing relevant items, and well-organized (see **Table 3**). No family found any questions distressing, while 4 healthcare professionals (8.9%) expressed discomfort with questions regarding partner relationships of the children’s parents.

**Table 3:** The analysis of the evaluation questionnaire.

		N	Mean	SD
Parents	How would you rate the length of the questionnaire? ^a^	48	3.2	0.6
	How comprehensible is the questionnaire? ^b^	48	1.9	0.7
	How relevant are the included items for pediatric palliative care? ^c^	48	1.9	0.8
	How do you evaluate the structure of the questionnaire form? ^d^	48	1.9	0.6
Professional caregivers	How would you rate the length of the questionnaire? ^a^	45	3.0	0.7
	How comprehensible is the questionnaire? ^b^	45	1.8	0.5
	How relevant are the included items for pediatric palliative care? ^c^	45	1.5	0.6
	How do you evaluate the structure of the questionnaire form? ^d^	45	1.7	0.6

^a^Scale ranges from 1 (far too short) to 5 (far too long).

^b^Scale ranges from 1 (very comprehensible) to 5 (very incomprehensible).

^c^Scale ranges from 1 (all items are relevant) to 4 (no item is relevant).

^d^Scale ranges from 1 (very well structured) to 5 (very poorly structured).

In comparing symptom burden between families and healthcare professionals, notable differences were observed in levels of agitation, pain, and sleep problems. Families reported higher levels of these symptoms in their children compared to healthcare professionals. No significant differences were found for other symptoms like secretions, respiratory distress, convulsions, or spasticity (See **Table 4**).

**Table 4:** The evaluation of the children’s symptom severity in parents and professional caregivers.

Symptoms	Parents mean, (SD) ^a^	Professional caregivers mean (SD) ^a^	p
Secretions	3.7 (1.6)	3.3 (1.2)	0.18
Respiratory distress	3.3 (1.8)	3.2 (1.6)	0.73
Agitation	3.5 (1.4)	2.9 (1.2)	0.03
Pain	3.3 (1.6)	2.6 (1.2)	0.02
Sleep problems	3.6 (1.6)	2.8 (1.3)	<0.01
Convulsions	2.4 (1.6)	2.3 (1.2)	0.62
Spasticity	3.3 (1.8)	3.0 (1.4)	0.44

^a^Scale ranges from 1(not present) to 6 (very pronounced).

In comparing five scales between families and healthcare professionals, notable difference was observed in the scale “coping with the disease”. Families reported higher level of coping with the disease compared to healthcare professionals. No significant differences were found for other remaining four scales (See **Table 5**).

**Table 5:** The evaluation of the scales in parents and professional caregivers.

Scales ^a^	Parents mean, (SD) ^b^	Professional caregivers mean (SD) ^b^	p
Child’s social participation (3 items)	3.5 (1.3)	3.8 (0.9)	0.27
Normalcy (5 items)	3.4 (1.4)	3.4 (1.1)	0.95
Social support ^c^ (3 items)	3.8 (1.0)	3.5 (0.9)	0.17
Coping with the disease ^c^ (3 items)	4.4 (1.1)	3.8 (1.0)	<0.01
Caregiver competencies (3 items)	4.5 (1.3)	4.4 (1.0)	0.62

^a^Each scale score was calculated by summing the total item scores and dividing the total by the number of items in the respective scale.

^b^Scale ranges from 1 (completely disagree) to 6 (completely agree).

^c^Two items in each of these scales were reverse-coded.

### Validity and reliability of the FACETS-OF-PPC

The analysis was conducted on a total of 102 responses, combining data from both parents and professional caregivers. Despite the extended sample size, the results indicated that the overall model fit was inadequate. The X²/df was 2.29, which is borderline acceptable but doesn’t reach the ideal threshold of less than 2. The other fit indices also fell short of common standards, with a CFI of 0.805 and a TLI of 0.757. The SRMR was 0.109, and the RMSEA was 0.114.

Internal consistency analysis using McDonald’s ω showed the following results: 0.51 for child’s social participation, 0.87 for normalcy, 0.20 for social support, 0.50 for coping with the disease, and 0.86 for caregiver competencies. The child’s social participation, social support, and coping with disease subscales had low internal consistency, while normalcy and caregiver competencies had adequate reliability.

### Discussion

The FACETS-OF-PPC is a family-centered, multidimensional outcome measure developed and validated in Germany, specifically for patients with severe neurological impairments and their families. This group represents the largest demographic in PPC, yet there is a significant gap in outcome measures tailored to their specific needs. Another important point about this theme is that, neurological disorders are closely linked to childhood cancer, not only with primary malignant central nervous system (CNS) tumors being the second most common malignancies in children (19), but also with CNS metastasis of hematologic cancers. These CNS tumors are the leading cause of death from childhood cancer and often involve significant neurological impairment throughout the disease trajectory. This makes neurological impairment a key focus for PPC teams working with children with cancer as well. Our study highlights that the Turkish version of FACETS-OF-PPC demonstrates insufficient validity and reliability. To better understand these shortcomings, we have structured our discussion into three main areas: demographic and descriptive analysis, validity of the Turkish version of FACETS-OF-PPC, and PC systems context.

### Demographic and descriptive analysis

Data from 51 children, their parents, and caregivers reflect the high complexity and care needs of PPC in Türkiye. Most children required extensive medical support, with 76.5% depending on some form of respiratory assistance and 86.3% relying on nutritional support, highlighting the severe health conditions common within this population. The high use of medical technology described in our study is much higher than other studies. Feudtner et al. reported tracheostomy rate as 10.1% and mechanical ventilator rate as 8.5% in their multi-center prospective study (12). The high rate of medical technology dependence in our cohort may be the result of the lack of legal basis of withholding or withdrawing of life-sustaining treatments in Türkiye (20, 21). This fact shapes the PC philosophy in countries. In the absence of clear end-of-life (EOL) decision-making codes, healthcare professionals often perform all possible invasive interventions, leading to higher technology dependence in our PPC units. Western countries have improved the EOL concept and they have established its ethicolegal framework (22, 23). The FACETS-OF-PPC originates from Germany, where PPC is supported by well-established standards, as outlined by Benini et al. (10). These standards provide an ethico-legal foundation for EOL decision-making and ensure consistent care across healthcare systems. Their work underlines the necessity of preparing families for EOL care through structured, transparent discussions that respect cultural and individual preferences. Moreover, they advocate for interdisciplinary collaboration to ensure that care decisions, including EOL planning, prioritize the best interests of the child, while balancing the complex dynamics between healthcare providers and families (10). In contrast, Türkiye’s PPC framework is still evolving, with limited formal guidelines addressing EOL care. New aspects and discussions about EOL concept are urgently needed in Türkiye, as well. Clear EOL decision-making codes, could help strengthen Türkiye’s PPC infrastructure while respecting local cultural contexts.

Families and caregivers found the FACETS-OF-PPC to be well-structured and relevant as indicated by Pelke et al. (11, 13). Yet healthcare professionals expressed slight discomfort with items related to family dynamics. This discomfort may not only reflect the inherent complexities of addressing familial and cultural factors but may also stem from a broader lack of communication skills among healthcare providers. Effective communication in the context of complex condition such as neurodevelopmental disorders and oncological disorders in Turkey requires sensitivity to cultural and spiritual dimensions, particularly when discussing sensitive topics such as a child’s illness or caregiving responsibilities (24). Poor communication skills among healthcare professionals can exacerbate stress and misunderstandings within families, potentially leading to reduced treatment adherence and emotional resilience.

The discrepancy between parents’ and caregivers’ reports of symptom burden is notable. In particular, parents reported higher levels of agitation, pain, and sleep disturbances in their children compared to healthcare professionals. This reflects findings from similar studies where healthcare providers underestimate the symptom intensities of the patients (25–27). However, reports on more objective symptoms like secretions and respiratory distress were consistent between observers. While parental reports provide critical insight into their child’s well-being, it is also possible for parents to overestimate symptom intensities due to heightened emotional distress or anxiety. Research indicates that parents of chronically ill children often report higher levels of perceived vulnerability and symptom severity, which may be influenced by their psychological state rather than objective symptomatology (28). These factors underline the importance of balancing parental observations with clinical assessments to ensure accurate evaluation and management.

### Validity of the Turkish version of FACETS-OF-PPC

This study revealed limitations in the overall model fit of the FACETS-OF-PPC, with fit indices failing to meet the accepted standards. Similar challenges were reported in Pelke et al.’s validation studies (11, 13), which also met difficulties in achieving optimal model fit for PPC populations. The inadequate internal consistency in scales such as social participation, social support, and coping with disease highlights the complexity of accurately measuring these dimensions in PPC. The inadequate model fit and low reliability of certain subscales may be attributable to several factors. First, the small sample size likely limited the statistical power of the CFA, contributing to the suboptimal fit indices. Moreover, the variability in the parent and professional caregiver groups, which were pooled for this analysis, could have introduced additional complexity that the current factor structure did not fully capture. The low internal consistency for social support and coping with the disease suggests that these constructs may be more heterogeneous than originally anticipated, or that the items within these subscales may not be fully capturing the intended dimensions of care within Turkish specific cultural context.

### Discussion of results in context of palliative care systems

The observed inconsistencies in internal reliability, particularly in the scales for child’s social participation, social support, and coping with the disease, may not just reflect issues with the psychometric properties of the Turkish version of the FACETS-OF-PPC scale. These results could also be indicative of broader structural and philosophical differences in the PC systems of the two countries. In contrast to the well-established PC frameworks observed in many Western nations, Türkiye’s PC philosophy is still in the process of developing, and, in some cases, the core principles of PC may not be fully set in into the healthcare system (5). This discrepancy could contribute to the lower internal consistency seen in key domains of the scale, as the organizational and philosophical approaches to PC differ significantly between countries. In Türkiye, where the PC model is still developing, certain areas may not be fully supported in practice or perception. This could influence how families and healthcare providers respond to questions on the scale. The structure and delivery of PC services in Türkiye might affect how care is viewed and measured, potentially explaining discrepancies in the internal consistency of the scale. Therefore, it’s important to consider both the tool’s limitations and the broader healthcare system when interpreting these results. More research is needed to explore how different PC approaches affect care measurements in various cultural and healthcare settings.

## Conclusion

Given these findings, it is clear that further revision and refinement of the Turkish version of the FACETS-OF-PPC are essential. Future studies should involve larger, more diverse samples and consider cultural adaptations to improve validity and reliability across populations. While the FACETS-OF-PPC shows promise as a multidimensional tool for PPC, ongoing refinement is needed to ensure psychometric robustness, particularly in diverse healthcare settings where PC philosophies may differ.

## Data Availability

The original contributions presented in the study are included in the article/supplementary material. Further inquiries can be directed to the corresponding author/s.
